# X-Shaped Dual-Band Slot Antenna with Simultaneous Pattern Diversity and Frequency Tuning

**DOI:** 10.3390/s26031047

**Published:** 2026-02-05

**Authors:** Youngjin Cho, Youngje Sung

**Affiliations:** Department of Electronic Engineering, Kyonggi University, Suwon 16227, Republic of Korea; cyjinstar@kgu.ac.kr

**Keywords:** beam switching, frequency tunable, pattern diversity, reconfigurable antenna, X-shaped slot

## Abstract

This paper proposes a frequency-reconfigurable and active beam-switching antenna based on an X-shaped slot array integrated with a diode-based switching network. The proposed antenna features four slots arranged at 90° intervals around the feed point. Each slot is integrated with two PIN diodes and one varactor diode. By selectively activating a specific slot through the PIN diodes, the radiation pattern can be switched in four directions at 90° intervals. Dual-band operation is achieved using varactor diodes, and by controlling their equivalent capacitance, the antenna covers two operating bands: a low-frequency band with a 29.51% bandwidth (2.6–3.5 GHz) and a high-frequency band with a 24.52% bandwidth (3.65–4.67 GHz). These frequency ranges include the 5G sub-6 GHz bands, specifically n77 and n78. Experimental results confirm stable beam-switching performance across the entire operating frequency range.

## 1. Introduction

Wireless sensor platforms and IoT devices demand compact and versatile antennas that can adapt their radiation characteristics and maintain reliable communication performance under varying spectral and environmental conditions [1]. Accordingly, antennas capable of supporting multiple beam patterns and operating frequencies in a selective manner within a single structure have received considerable attention. In particular, pattern diversity antennas, which enable beam steering without complex arrays, offer advantages in hardware simplicity and circuit integration. Pattern-reconfigurable antennas are particularly attractive because they can steer or redirect the radiation coverage without relying on large or complex antenna arrays, which makes them suitable for low-profile sensing modules and embedded wireless nodes.

To address these requirements, various multi-slot antenna structures capable of selective beam steering have been proposed. In [2], a reconfigurable antenna using four tapered slot antennas (TSAs) and a tunable feeding network achieves pattern reconfiguration with eight beam directions spaced at 45° intervals. In a similar approach, an antenna structure employing three tightly spaced slots arranged in eight directions was proposed to achieve eight beam directions [3]. Another design uses four U-shaped slots to realize eight distinct beam directions as well [4]. The work in [5] presents a design with two meandered slots and a monopole to realize four beam directions. All these designs implement beam-switching functionality using reconfigurable feeding networks based on PIN diodes.

As previously described, most existing designs are based on slot antennas that offer a wide beam-switching range. However, these studies mainly emphasize radiation pattern steering, while frequency reconfigurability for adapting to dynamically changing spectral environments has attracted relatively little attention [2,3,4,5,6,7]. Most of these structures employ open-ended slots, such as TSA, in which the resonance behavior is only weakly influenced even with the introduction of varactor diodes. Consequently, effective frequency tuning is difficult to achieve, and continuous frequency reconfiguration is generally not supported. Some designs allow switching among several predefined frequency bands, but they still do not provide continuous frequency tuning [8]. In contrast, antenna structures that achieve continuous frequency tuning typically lack beam-switching capability [9,10].

The proposed structure employs a close-ended slot configuration, enabling the integration of adaptive beam-switching and continuous frequency-tuning functionalities within a single antenna. These characteristics are well suited for 5G NR-based wireless sensor platforms operating in dynamic propagation environments, where link reliability is highly dependent on device orientation and spectral conditions. In industrial IoT scenarios featuring non-line-of-sight propagation and time-varying interference, beam switching improves link robustness through adaptive control of the radiation pattern, while continuous frequency tuning enables flexible operation across available sub-6 GHz bands. These functionalities are realized within a compact antenna structure, providing a hardware-efficient solution for sensor nodes deployed in complex wireless environments. Furthermore, the proposed reconfigurable antenna is suitable as a potential shared frontend for emerging sensing-and-communication platforms that require simultaneous spatial and spectral adaptability.

This paper proposes an X-shaped dual-band slot antenna capable of both pattern reconfiguration and continuous frequency tuning, covering the sub-6 GHz bands, including 5G NR bands n77 and n78. The proposed antenna consists of four intersecting slots arranged on a 60 × 60 mm^2^ substrate, with each slot integrated with two PIN diodes and one varactor diode. By configuring the ON/OFF states of eight PIN diodes, four directional radiation patterns—aligned at 0°, 90°, 180°, and 270° in the xy-plane—are achieved, enabling beam switching at 90° intervals. Simultaneously, continuous frequency reconfiguration is realized in two bands, 2.6–3.5 GHz and 3.65–4.67 GHz, by adjusting the equivalent capacitance of the varactor diodes.

The remainder of this paper is organized as follows: Section 2 describes the proposed X-shaped slot structure and the mechanisms for frequency and pattern reconfiguration. Section 3 analyzes the parametric effects on antenna performance. Section 4 presents the biasing circuitry and compares simulated and measured results. Finally, Section 5 concludes the paper with a summary of the key findings.

## 2. Antenna Design and Analysis

### 2.1. Antenna Configuration

**Figure 1:**
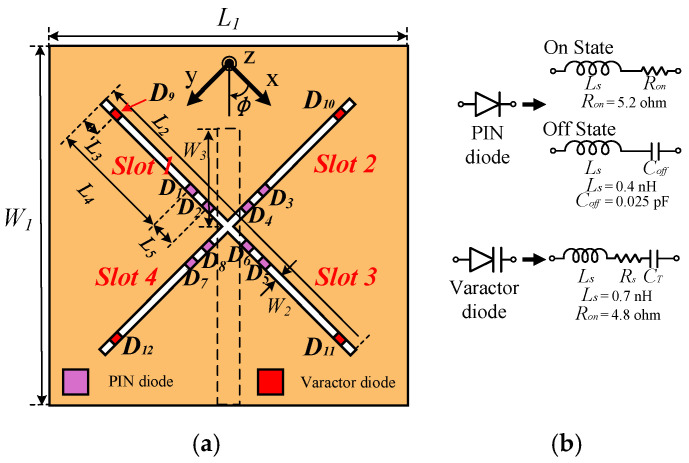
(**a**) Basic structure of the proposed antenna. (**b**) Equivalent circuit model of the diode used.

Figure 1a illustrates the structural configuration of the proposed slot antenna. The antenna was fabricated on a Taconic TLY-5 substrate manufactured by SungJin Flontec Co., Ltd., Incheon, Republic of Korea, with a relative permittivity of 2.2 and a thickness of 31 mil. The TLY-5 laminate exhibits a low loss tangent of approximately 0.0009 at 10 GHz, which minimizes dielectric loss and supports efficient RF performance in the sub-6 GHz band. To efficiently arrange four slots (Slot 1–Slot 4), the resonating structure adopts an X-shaped configuration. Each slot is loaded with two PIN diodes (shown in purple) and one varactor diode (shown in red). Figure 1b presents the equivalent circuit models of the PIN and varactor diodes used in the antenna design. The antenna is fed by a microstrip line located on the backside of the substrate, which is indicated as a dashed line in Figure 1a.

The four slots are selectively activated by controlling the ON/OFF states of the eight PIN diodes (D_1_–D_8_, purple). At the end of each slot, a varactor diode (D_9_–D_12_, red) is mounted to serve two key functions. First, the placement of the varactor near the center of the slot enables dual-band operation [11]. Second, by varying the equivalent capacitance of the varactor, the two resonant frequencies of the slot can be tuned, allowing for flexible control over the operating frequency bands.

### 2.2. Working Principle

#### 2.2.1. Beam Tilting

**Figure 2:**
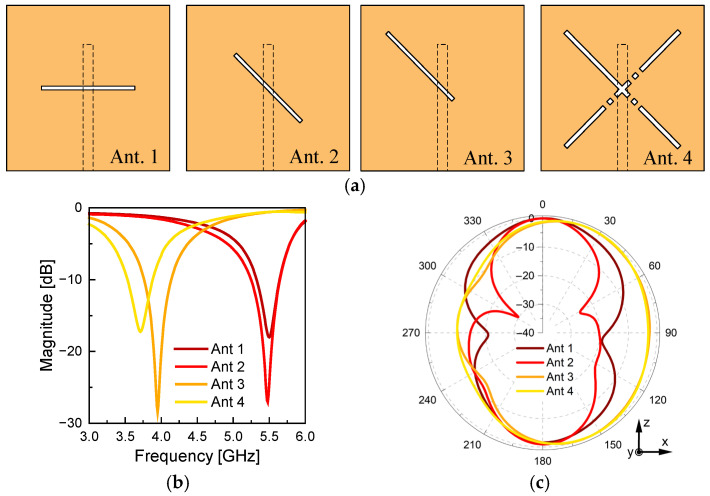
Antennas with different slot placements. (**a**) Configuration. (**b**) Simulated reflection coefficient. (**c**) Simulated radiation pattern.

Figure 2 compares four antenna structures (Ant. 1–Ant. 4) with different slot arrangements and positions while maintaining a fixed microstrip feed point. In Ant. 1, the slot is horizontally aligned, and the feed point is located exactly at the center of the slot. Ant. 2 features a 45° rotated slot with the feed point still placed at the center, identical to Ant. 1 in terms of feed positioning. Ant. 3 maintains the 45° rotation of Ant. 2 but relocates the feed point from the center to the end of the slot.

Compared to Ant. 1 and Ant. 2, Ant. 3 exhibits two major differences. First, the resonant frequency shifts significantly from approximately 5.5 GHz to 4.0 GHz. This is attributed to the increased effective electrical length caused by relocating the feed to the slot end and the reduced input impedance of the tilted slot, which brings it closer to its magnetic resonance frequency [12]. Second, the radiation pattern becomes clearly tilted in a specific direction. This directional skew results from the asymmetric feed location near the slot edge, which alters the current distribution and phase characteristics, thereby steering the radiation beam toward one side.

In addition, due to the structural symmetry of Ant. 3, its performance remains consistent regardless of the orientation—whether it is placed upper-left, upper-right, lower-left, or lower-right. Based on the efficient utilization of the slot configuration, a four-slot structure combining four identical Ant. 3 elements is determined to be optimal in an X-shaped cross arrangement, leading to the design of Ant. 4, only the upper-left slot activated among the four. 

The proposed antenna operates in four modes (Mode 1–Mode 4), where only one of the four slots is activated at a time while the others remain deactivated. For example, in Mode 1, only Slot 1 is active while Slots 2–4 are disabled. This is achieved by setting the PIN diodes (D_1_, D_2_) associated with Slot 1 to the OFF state and the remaining diodes (D_3_–D_8_) to the ON state. As a result, only Slot 1 radiates through the central microstrip feed line, realizing the characteristics of a 45°-tilted, offset-fed, varactor-loaded slot antenna without interference from the other slots. Due to the symmetric X-shaped configuration of the antenna, the same operating principle applies to Modes 2–4, where each of the other slots (Slot 2, Slot 3, and Slot 4) is activated individually. This structural symmetry ensures that all four modes exhibit equivalent radiation and frequency-tuning characteristics.

**Figure 3:**
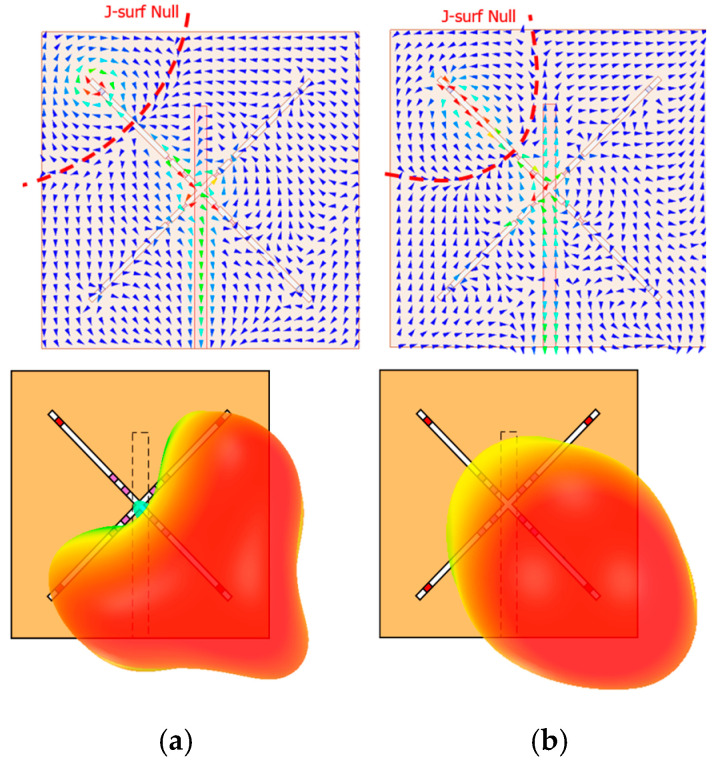
Simulated surface currents and corresponding 3-D radiation patterns at (**a**) 2.8 GHz and (**b**) 4.21 GHz.

Figure 3 illustrates the simulated surface current distributions and radiation patterns at the two resonant frequencies, *f*_L_ (2.8 GHz) and *f*_H_ (4.21 GHz), when operating in mode 1. At this time, the varactor diode D_9_ is set to 0.81 pF. The antenna parameters used in the simulation are as follows: *W*_1_ = 60 mm, *W*_2_ = 1 mm, *W*_3_ = 16 mm, *L*_1_ = 60 mm, *L*_2_ = 59 mm, *L*_3_ = 3 mm, *L*_4_ = 21 mm, and *L*_5_ = 4.24 mm. In Figure 3, the surface current nulls are indicated by red dashed lines. As observed, the null regions are offset from the geometric center of the X-shaped slot and are asymmetrically distributed relative to the feed structure, resulting in an asymmetric current distribution and a phase imbalance. This current skew causes the main radiation beam to tilt in the direction of the current null, rather than aligning with the structural center. Consequently, a beam tilt of approximately 45° is observed. In slot antenna structures, it is well known that asymmetric placement of current nulls leads to tilted beam radiation [13,14].

#### 2.2.2. Dual-Band Operation

**Figure 4:**
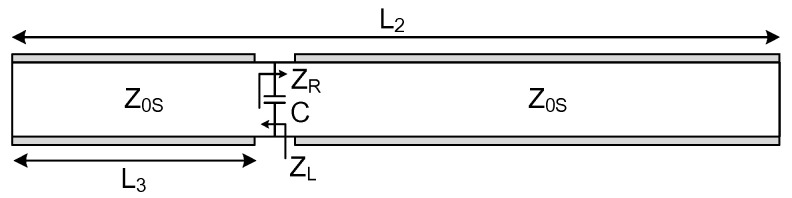
Transmission line model of a slot antenna loaded with a lumped capacitor.

A slot antenna is often modeled as an equivalent transmission-line circuit [11]. As depicted in Figure 4, placing a varactor diode at the open end introduces an increase in the line’s capacitance, which in turn lowers the resonant frequencies of both the fundamental and second-order modes. Notably, this frequency shift is not uniform—it varies according to the varactor’s position, its capacitance value, and the inherent characteristic impedance of the slot line [15].

The resonance frequencies can be determined by applying the transverse resonance condition [16]

(1) $$Z_{R}+Z_{L}=0$$

where *Z_R_* and *Z_L_* are the input impedances to the right and left of the reference point, as shown in Figure 4, and can simply be obtained from the following equations

(2) $$Z_{R}=\frac{jZ_{0S}\tan\left(\theta -\theta_{1}\right)}{1-\omega CZ_{0S}\tan\left(\theta -\theta_{1}\right)}$$

(3) $$Z_{L}=j\tan\left(\theta_{1}\right)$$

(4) $$\theta =\beta \left(\omega \right)L,\;\theta_{1}=\beta \left(\omega \right)L_{1}$$

Here, *β* denotes the propagation constant along the slot line, *ω* is the angular frequency, *C* represents the varactor diode’s capacitance, *Z*_0*S*_ is the characteristic impedance of the slot line, *L* is the overall slot length, and *L*_1_ designates the varactor’s distance from the slot’s open end. Substituting expressions (2) and (3) into Equation (1) yields the resonance condition:

(5) $$\tan\left(\theta -\theta_{1}\right)+\tan\left(\theta_{1}\right)-\omega CZ_{0s}tan\left(\theta -\theta_{1}\right)\tan\left(\theta_{1}\right)=0$$

**Figure 5:**
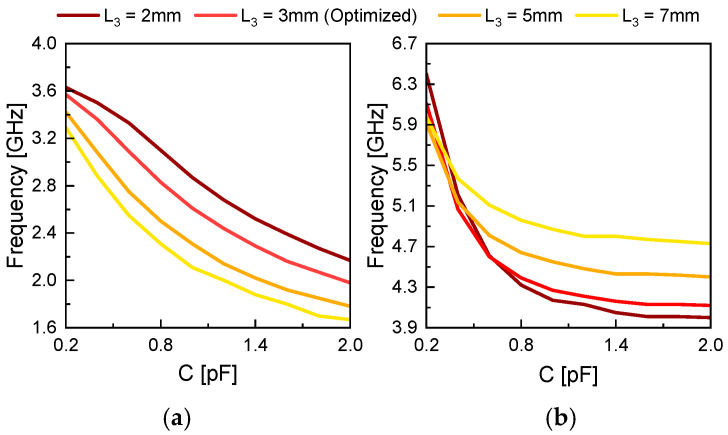
Resonant frequencies of a microstrip-fed loaded slot antenna with *L*_2_ = 59 mm and *W*_2_ = 1 mm. (**a**) First resonant frequency. (**b**) Second resonant frequency.

Figure 5 shows the variation in the first and second resonant frequencies with respect to the change in varactor diode position, *L*_3_, when the total slot length is fixed at *L*_2_ = 59 mm and the slot width is *W*_1_ = 1 mm. The propagation constant and impedance of the slot were calculated using the equations presented in [17]. As the capacitance *C* of the varactor diode increases, both resonant frequencies decrease. This behavior is attributed to the increased effective capacitance of the slot caused by the parallel loading of the varactor, which shifts the resonance condition toward lower frequencies. As the value of *L*_3_ increases—i.e., as the varactor diode moves closer to the end of the slot—the resonant frequency curves shift further into the lower frequency range. This shift is due to changes in the current path and impedance distribution of the slot between the diode and the feed point, thereby tuning the resonant characteristics. Hence, Figure 5 demonstrates that by simultaneously adjusting both the capacitance and position of the varactor diode, a dual tuning mechanism is enabled for precise control of the dual-band resonant frequencies.

**Figure 6:**
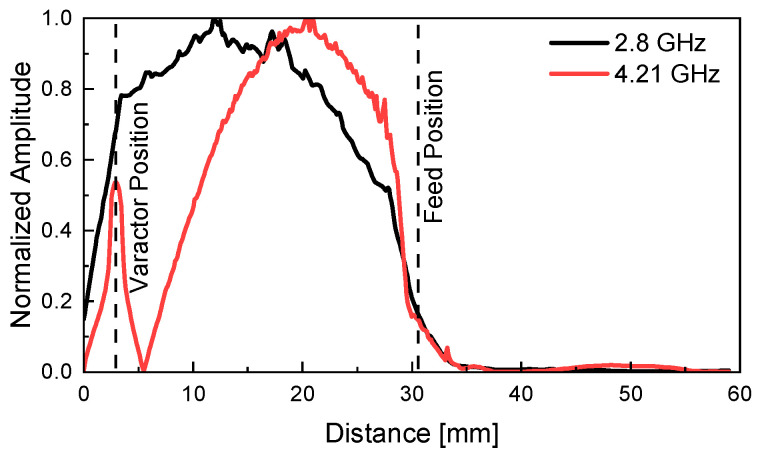
The simulated normalized E-field distribution along the slot surface.

Figure 6 presents the simulated distribution of the normalized electric field (E-field) magnitude along the slot surface when only Slot 1 is active. In Figure 6, the variable distance denotes the position measured from the outer end of Slot 1; that is, 0 mm corresponds to the outer end of Slot 1, while 59 mm corresponds to the outer end of Slot 3. The simulation results confirm that beyond the feed point at 30.5 mm, the E-field distribution is negligible in the Slot 3 region. Moreover, based on the E-field distributions, it is evident that both resonant modes at *f*_L_ = 2.8 GHz and *f*_H_ = 4.21 GHz exhibit a typical half-wavelength (λ/2) resonance behavior.

At *f*_L_, the varactor diode presents a relatively small capacitance, preventing RF power from passing through it. As a result, resonance occurs across the full slot length. In contrast, at *f*_H_, the RF signal effectively passes through the varactor diode, behaving as if the slot is short-circuited at the diode’s position. This creates a reduced effective slot length by approximately *L*_3_, leading to a shorter resonant structure.

## 3. Parameter Study

**Figure 7:**
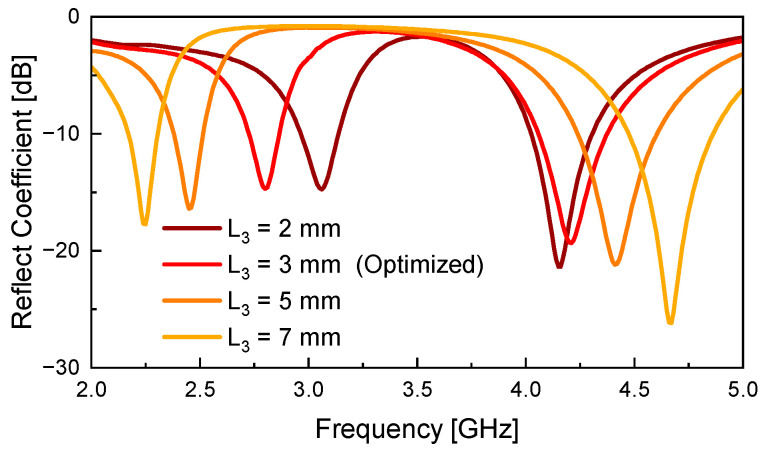
Simulated *S*_11_ results as a function of the varactor diode position *L*_3_.

Figure 7 illustrates the simulated *S*_11_ responses, demonstrating how the resonant frequencies *f*_L_ and *f*_H_ shift as a function of the varactor diode position *L*_3_. All simulations are performed with the varactor diode’s equivalent capacitance fixed at 0.81 pF. At *f*_L_, the capacitance of the varactor is not sufficiently large to allow RF current to pass through, so the diode appears as an open circuit. In this case, it effectively increases the electrical length of the slot. As *L*_3_ increases, the diode moves closer to the center of the slot, where the electric field is strongest, leading to a decrease in *f*_L_.

On the other hand, at *f*_H_, the diode acts as a short-circuit point along the slot. As the diode moves further from the slot end, the effective slot length is reduced, causing *f*_H_ to increase. As a result, the frequency ratio (*f*_H_/*f*_L_) exhibits an approximately linear increase with *L*_3_, and remains within a deviation of about 0.34% from the theoretical value given in (6) over the range of *L*_3_ = 2–7 mm.

(6) $$\frac{f_{H}}{f_{L}}=1.067+0.145L_{3}$$

When *L*_3_ ≤ 3 mm, the shift in *f*_H_ becomes negligible, which may affect the frequency tunability of the proposed structure. Therefore, *L*_3_ = 3 mm is finally selected as the optimal position that ensures minimal impact on frequency tuning performance while maintaining a sufficiently small spacing between the two resonant frequencies.

**Figure 8:**
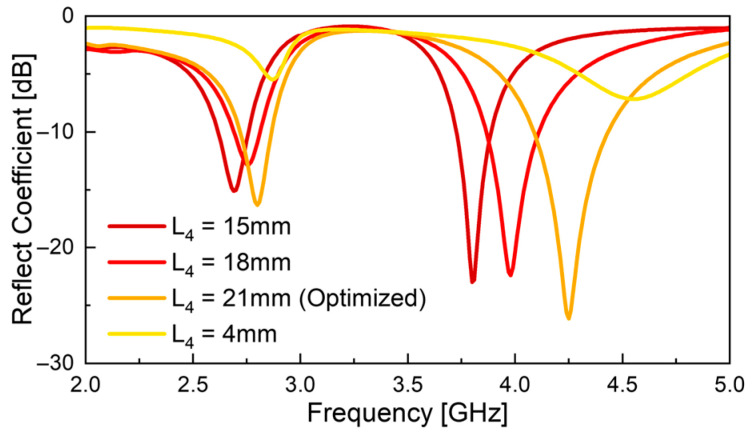
Simulated *S*_11_ responses with respect to the PIN diode position *L*_4_.

Figure 8 illustrates the variation in antenna performance as a function of the decrease in PIN diode position *L*_4_—i.e., as the diode moves closer to the center of the slot, the effective length of the active slot increases, resulting in a downward shift in the overall resonant frequency. However, this trend does not affect both resonant frequencies equally. In the low-frequency band, the frequency shift is relatively small, whereas in the high-frequency band, the change in *L*_4_ leads to a more pronounced shift in the resonant frequency.

At *f*_L_, the varactor diode operates electrically as an open circuit, so the entire slot, including the region containing the varactor, contributes to resonance. Due to the varactor’s capacitance, the slot exhibits an electrically longer length than its physical length of 33.76 mm. For instance, assuming the slot operates as a λs/2 resonator, the calculated half-wavelengths λs/2 based on (7) are approximately 49.55 mm for the low-frequency band and 31.85 mm for the high-frequency band. Consequently, the same physical change in length represents a larger fractional variation in the slot length at *f*_H_, making the resonant frequency more sensitive to changes in *L*_4_ in the higher band.

(7) λs=λ0[1.045−0.365ln εr+6.3Whεr0.945238.64+100Wh−0.148−8.81εr+0.95100εr·lnhλ0

**Figure 9:**
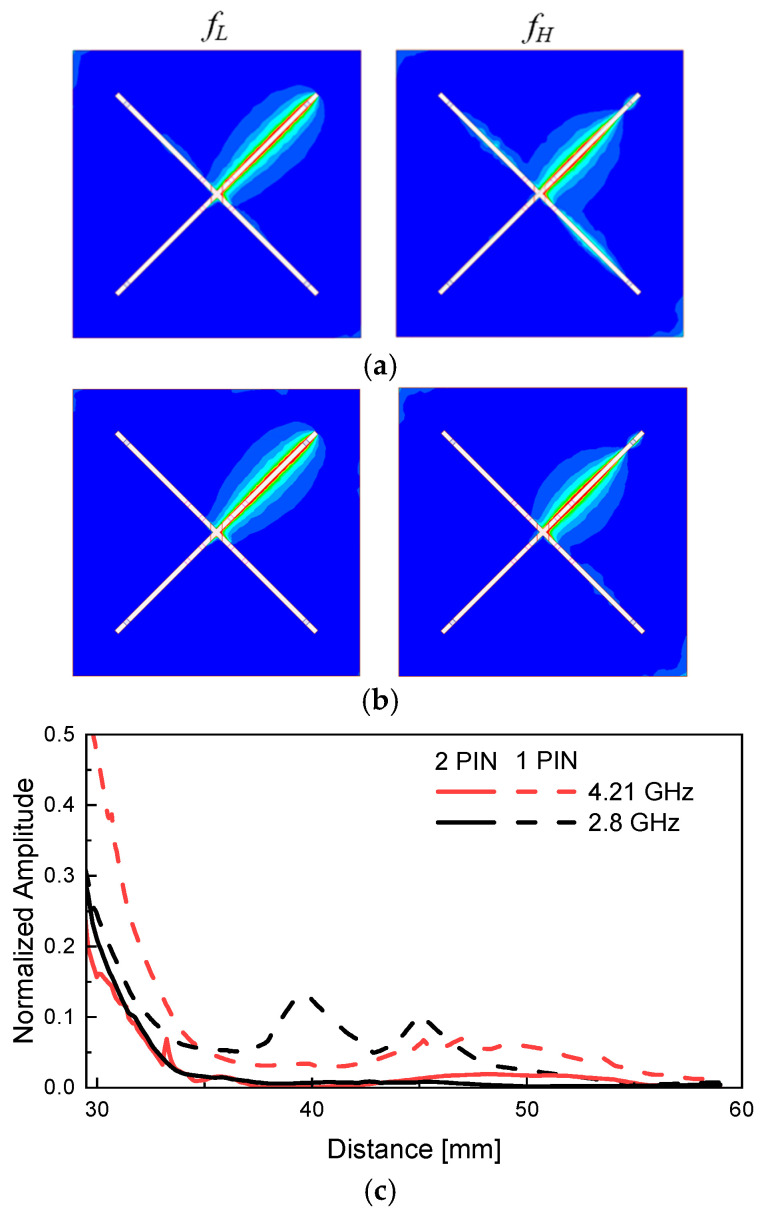
Simulated electric field distribution at both *f*_L_ and *f*_H_. (**a**) When one PIN diode is used. (**b**) Two PIN diodes are used. (**c**) normalized amplitude of a closed slot.

Figure 9 presents the simulated E-field distributions at the lower frequency band *f*_L_ and the higher frequency band *f*_H_, according to the number of PIN diodes integrated per slot. Figure 9a illustrates the E-field distribution when one PIN diode is placed per slot, while Figure 9b shows the case where two PIN diodes are used per slot.

When only one PIN diode is used per slot, activating a specific slot in the open state results in field leakage not only in the intended slot but also in adjacent slots. This phenomenon indicates insufficient isolation between the slots, allowing electromagnetic energy excited in the target slot to couple into neighboring slots. Such leakage deteriorates beam control capability and reduces the overall directivity performance of the antenna. Figure 9c shows the normalized electric field magnitude along the slot surface using the same method as in Figure 6. The plotted range is enlarged to focus on Slot 3 in Figure 6. The black and red curves correspond to the electric field magnitude at *f*_L_ and *f*_H_, respectively. The solid lines indicate the case with two PIN diodes per slot, while the dashed lines represent the case with one PIN diode per slot. The electric field magnitude observed in the unexcited slot is approximately 14 times larger at *f*_L_ and 3.57 times larger at *f*_H_ when only one PIN diode is used, compared to the case with two PIN diodes. These results quantitatively demonstrate that employing two PIN diodes per slot effectively suppresses unwanted excitation and improves inter-slot isolation.

In contrast, when two PIN diodes are implemented per slot, the E-field distribution is well confined to the activated slot, with minimal field presence in the adjacent slots. This implies a clearer distinction between the open and short states of each slot, significantly improving inter-slot isolation. The enhanced isolation achieved by employing two PIN diodes per slot effectively suppresses unwanted current leakage and greatly improves the precision and reliability of the proposed beam-switching mechanism by enabling selective activation of a single slot. As a result, the configuration with two PIN diodes per slot provides accurate control over the resonant state of each slot and demonstrates that the proposed antenna can effectively and stably form a directional beam toward one of the four designated directions.

## 4. Results and Discussion

**Figure 10:**
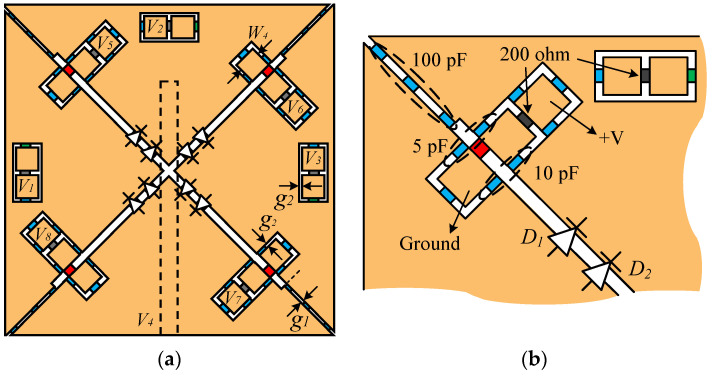
(**a**) Proposed antenna structure with integrated biasing circuit (**b**) A magnified view of the Slot 1 area.

Figure 10 presents the overall structure of the proposed X-shaped slot antenna with an integrated bias circuit. Figure 10a shows the antenna structure along with the positions of the bias circuits corresponding to each slot, while Figure 10b presents a magnified view of Slot 1, showing the detailed configuration of the circuit. The bias circuit consists of capacitors (blue), resistors (gray), and inductors (green). These passive components are used to stably apply bias voltages to each diode and to prevent mutual interference between high-frequency signals and the DC power supply.

All capacitors without indicated values are set to 39 pF, which is optimized for blocking high-frequency signals and isolating the bias line. The bias voltages for switching the PIN diodes are *V*_1_ through *V*_4_, and those for controlling the capacitance of the varactor diodes are *V*_5_ through *V*_8_. The voltage *V*_4_ is applied using a bias tee. To activate only one slot, a reverse bias is applied to the PIN diodes of the target slot, while a forward current is allowed to flow through the others. For example, to activate Slot 1, a positive voltage is applied to *V*_2_ and ground is connected to *V*_1_. This configuration enables selective operation of a specific slot to form a beam in the desired direction.

The bias voltages for the varactor diodes are applied through three bias pads located adjacent to each diode. As shown in Figure 10b, the rightmost pad receives a positive voltage, and the leftmost pad is connected to ground. Each pad is configured independently through separate capacitors, ensuring electrical isolation from the operation of the PIN diodes. This minimizes interference between the frequency tuning function and the beam-switching function.

**Figure 11:**
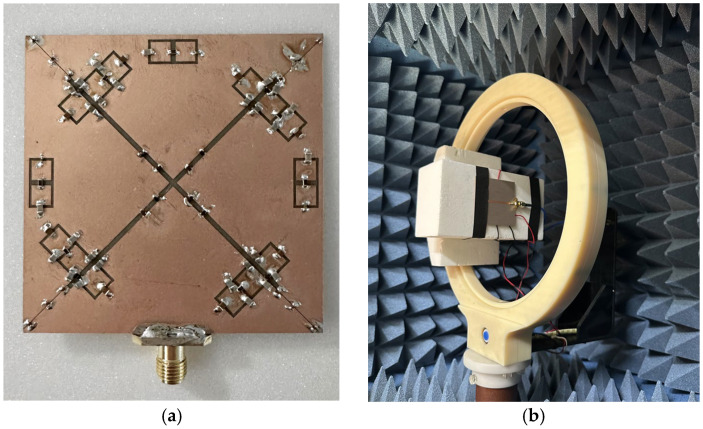
Photographs of the proposed antenna. (**a**) fabricated prototype of the proposed structure. (**b**) fabricated antenna under test.

The slot width *W*_4_ is set to 1.5 mm, with *g*_1_ and *g*_2_ set to 0.1 mm and 0.5 mm, respectively, for the inter-slot spacing and the bias circuit gap. The remaining variables follow the definitions given in the previous sections. Figure 11 presents photographs of the proposed antenna. Figure 11a shows the fabricated prototype of the antenna, while Figure 11b shows the antenna under test during the measurement in the anechoic chamber. The antenna characteristics are measured using the vector network analyzer E5071C, and the far-field radiation patterns are measured in a fully anechoic chamber (mmWK-1) provided by Korea Shield System, Ltd. The ON/OFF states of the PIN diodes for the four operating modes are summarized in Table 1.

**Figure 12:**
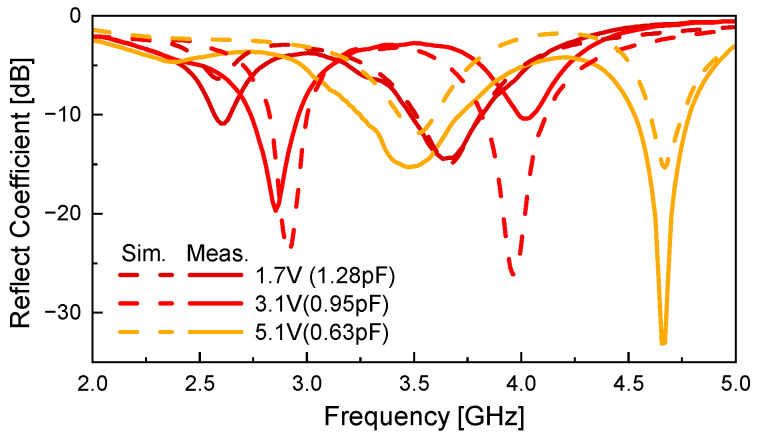
Comparison of simulated and measured reflection coefficients.

Figure 12 shows a comparison between the simulated and measured results of the antenna presented in Figure 10. Both simulation and measurement are conducted in Mode 1. The varactor diode is biased with voltages of 1.7 V, 3.1 V, and 5.1 V, corresponding to equivalent capacitances of 1.28 pF, 0.95 pF, and 0.63 pF, respectively. As the applied bias voltage increases, the lower resonant frequency *f*_L_ shifts from 3.5 GHz to 2.6 GHz, and the higher resonant frequency f_H_ shifts from 4.67 GHz to 3.65 GHz. This corresponds to fractional frequency tuning ranges of 29.5% and 24.5%, respectively, with respect to the center frequencies. Reasonable agreement is observed between the simulated and measured, validating the effectiveness of the proposed design.

**Figure 13:**
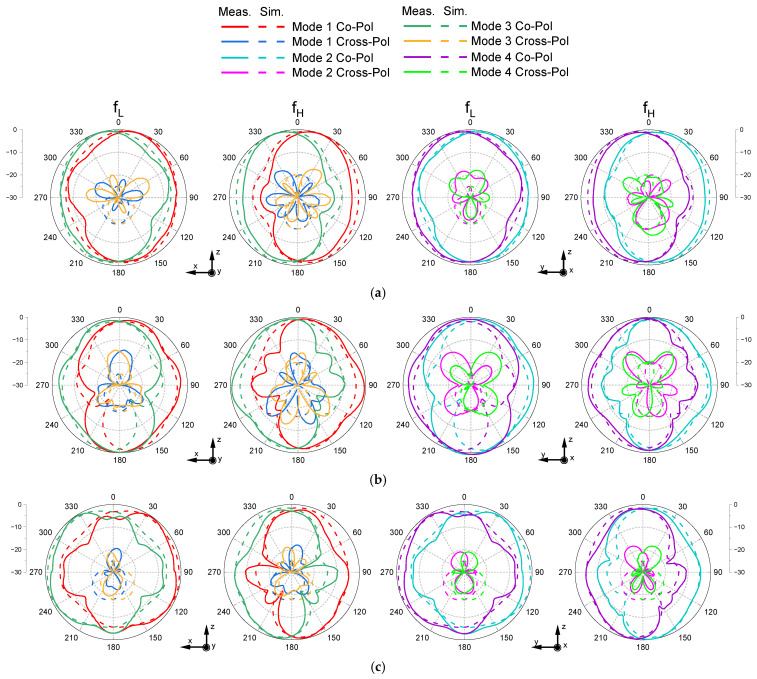
Simulated and measured radiation patterns at applied bias voltages of (**a**) 1.7 V, (**b**) 3.1 V, and (**c**) 5.1 V for each operating mode.

Figure 13 shows the simulated and measured radiation patterns for all four operating modes under the same bias conditions as in Figure 12. The radiation patterns are presented in the xoz-plane, where the maximum radiation occurs. Overall, the measured and simulated results exhibit consistent radiation characteristics, with a clearly observable unidirectional beam tilt under each condition. The measured co-polarized peak gain ranges from 1.16 to 2.61 dBi. To further examine the influence of the DC bias wires, they were included in the full-wave electromagnetic simulation. The comparison confirms that the presence of the bias wires has a negligible effect on the radiation pattern shape and the main beam direction. Therefore, the minor discrepancies observed between the simulated and measured results are more likely attributed to fabrication tolerances and assembly imperfections rather than the bias wires themselves.

**Figure 14:**
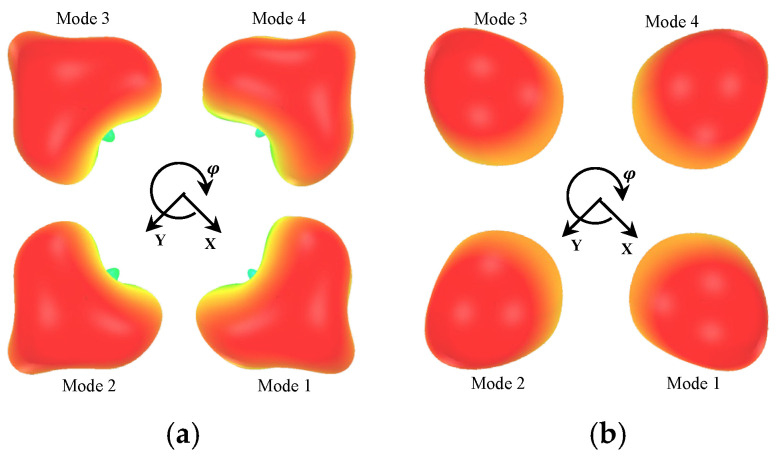
Simulated 3D radiation pattern. (**a**) at *f*_L,_ (**b**) at *f*_H_.

Figure 14 illustrates the simulated 3D radiation patterns of the proposed antenna structure for four different modes. Figure 14a and Figure 14b present the radiation characteristics at the lower frequency band (*f*_L_) and the higher frequency band (*f*_H_), respectively. Each mode corresponds to the radiation pattern when an individual slot is selectively activated, and it is evident that the radiation direction varies significantly depending on which slot is excited. These results demonstrate that the proposed antenna structure can generate distinct radiation directions, supporting its potential as a radiation pattern reconfigurable antenna.

**Figure 15:**
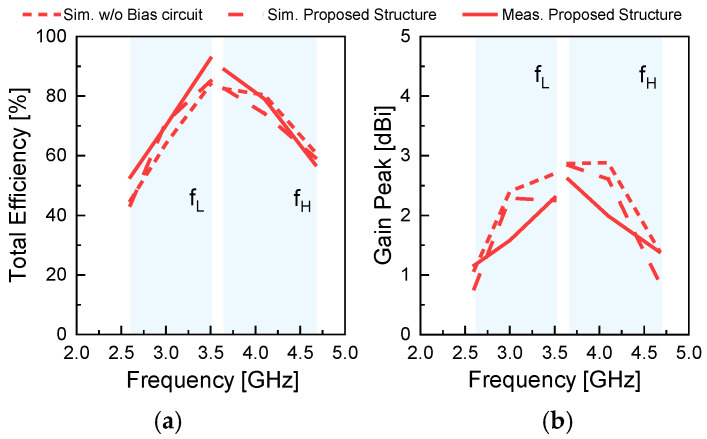
Simulated and measured performance of the proposed antenna: (**a**) total efficiency and (**b**) peak realized gain.

Figure 15 presents the simulated and measured performance of the proposed antenna in its fully integrated configuration, including all PIN diodes, varactor diodes, and biasing circuits. Figure 15a shows the total efficiency, while Figure 15b shows the peak realized gain under the same operating conditions. The total efficiency was calculated based on the measured antenna gain and the directivity obtained from the measured radiation patterns. To examine the impact of the biasing circuitry, simulations were performed with and without the bias network while maintaining the same radiating structure and active components.

As shown in Figure 15a, the total efficiency is not significantly affected by the bias circuit. The simulated total efficiencies with and without the bias network remain close over most of the operating frequency range. The measured total efficiency, obtained with the bias circuit implemented, ranges from 52.8% to 92.7% and follows the simulated trend across the operating band. Figure 15b shows the corresponding peak realized gain. A higher peak gain is observed in the simulation without the bias circuit over part of the operating band, while the measured peak gain follows the same overall trend as the simulated results. These results indicate that the bias circuit has a limited impact on the total efficiency, while it influences the peak gain level.

In the proposed antenna, the removal of the varactor diodes changes the resonance characteristics, and the resulting structure represents a different antenna rather than a reference case for efficiency comparison. As a result, the antenna without varactor diodes cannot serve as a valid reference for radiation-efficiency comparison. For this reason, the radiation efficiency of the proposed antenna is evaluated only for the configuration in which all diodes and biasing circuits are included.

**Figure 16:**
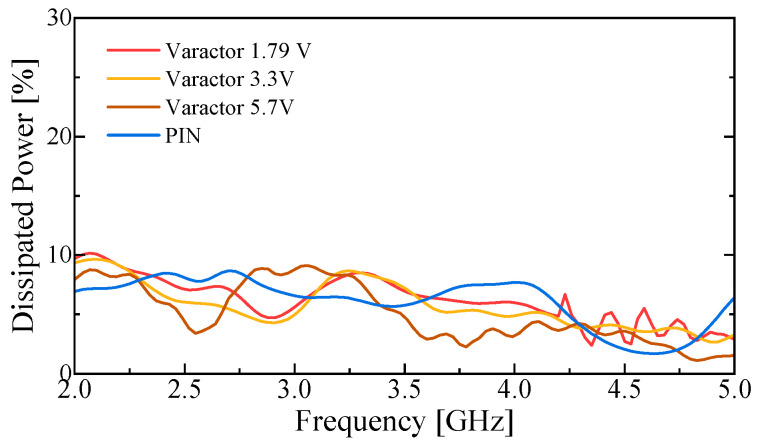
Measured dissipated power of the PIN and varactor diodes used in the proposed antenna under different bias conditions.

To assess the contribution of the reconfiguration elements to the observed efficiency reduction, the RF losses of the PIN diode and the varactor diode were characterized independently, as shown in Figure 16. Each diode was mounted at the center of a 50-Ω transmission line, and its scattering parameters were measured. The dissipated RF power was calculated as

(8) $$P_{diss}=1-{\left|S_{11}\right|}^{2}-{\left|S_{21}\right|}^{2}$$

The measured results indicate that both diodes introduce a certain level of RF power dissipation under the applied bias conditions. These measurements provide a quantitative reference for estimating the contribution of the diodes to the radiation efficiency degradation observed in Figure 15.

Table 2 compares the performance of the proposed antenna with that of previously reported designs. Radiation efficiency values are included only when they are explicitly reported or can be unambiguously derived from the referenced literature. For works where efficiency data are not provided, the corresponding entries are marked as “None”. Unlike conventional slot antenna structures focused on single functionality, the proposed design achieves both beam switching and continuous frequency tunability within a compact form factor.

Based on the experimental results, the proposed antenna demonstrates that simultaneous beam reconfiguration and continuous frequency tuning can be realized within a compact slot-based structure without relying on complex arrays or additional RF chains. The proposed reconfigurable structure offers several directions for further enhancement in practical deployments. In future work, the beam-switching capability can be extended to achieve finer angular resolution or full 360° radiation coverage by increasing the number of reconfigurable states or optimizing the slot excitation scheme. Such extensions would allow more flexible spatial control while preserving the compact form factor of the antenna. In addition, a further reduction in diode-related losses and refinement of the biasing network may improve radiation efficiency across the tuning range. Integration with practical RF frontends and sensor modules will also be explored to assess system-level performance under realistic operating conditions. Owing to its combined spatial and spectral adaptability, the proposed antenna may serve as a potential shared frontend for emerging sensing-and-communication platforms.

## 5. Conclusions

In this study, an X-shaped slot antenna capable of both pattern diversity and frequency reconfigurability is proposed. PIN diodes are employed to selectively activate one of the four slots, enabling four-directional beam switching at 90° intervals. Additionally, by varying the capacitance of varactor diodes, the antenna operates over two continuous frequency ranges. The corresponding operational bandwidths, defined by |S11| < −10 dB, are 36.31% and 31.48%, respectively. To ensure stable operation of both the PIN and varactor diodes, an optimized biasing network is designed to accurately supply the required bias voltages. The simulated and measured results demonstrate consistent impedance behavior and reliable beam-tilting characteristics across all operating modes, confirming that the proposed structure maintains stable performance under varying bias conditions. Owing to its compact geometry, directional control capability, and wideband frequency tunability, the proposed antenna can serve as a compact and adaptive RF front-end for sub-6 GHz 5G NR sensor nodes that require flexible spatial coverage and frequency agility without complex antenna arrays.

## Figures and Tables

**Table 1:** Applied bias voltage conditions and operating conditions.

Operating Mode	Condition [V]				Diode State [0: Off, 1: On]							
	*V*_1_	*V*_2_	*V*_3_	*V*_4_	D_1_	D_2_	D_3_	D_4_	D_5_	D_6_	D_7_	D_8_
1	0	3	0	0	0	0	1	1	1	1	1	1
2	0	0	3	0	1	1	0	0	1	1	1	1
3	0	0	0	3	1	1	1	1	0	0	1	1
4	3	0	0	0	1	1	1	1	1	1	0	0

**Table 2:** Comparison with previous works.

Ref.	Freq. [GHz]	BW [%]	Gain [dBi]	Frequency Tunability	Beam Switching Mode	Efficiency [%]	Size [λ_0_ × λ_0_]
[2]	4.79	68	3–5.2	None	8	81–93	0.51 × 0.51
[3]	10	24	4.95–5.45	None	8	None	0.88 × 0.88
[4] *	3.65	5.5	9.35	None	8	28.2–82.5	0.66 × 0.65
[5]	0.87	2.53	−3.5–1.58	None	4	43.3	0.23 × 0.159
[6]	0.8	25	9	None	5	None	0.8 × 0.8
[7]	5.79	3.2	7–8.65	None	4	64.4–88.3	1.92 × 0.76
[8]	3.5/5.5	7.1/15.9	0.85–1.11	Discrete	2	53	0.36 × 0.44
[9]	11.15	77.8	−4.4–−3.5	Continuous	None	None	0.35 × 0.7
[10]	2.1	41	5–6	Continuous	None	69.6–77.4	0.41 × 0.41
This work	3.14/4.13	36.3/31.5	1.16–2.61	Continuous	4	52.8–92.7	0.725 × 0.725

* Single element only.

## Data Availability

The raw data supporting the conclusions of this article will be made available by the authors on request.

## Abbreviations

The following abbreviations are used in this manuscript:

IoT	Internet of Things
DC	Direct Current
NR	New Radio
PIN	Positive-Intrinsic-Negative
E-field	Electric Field

## References

[1] Öner G.G.B., Başbuğ S., Altuncu Y. (2025). Design of a Compact Multilayer High-Gain Microstrip Patch Antenna for IoT Applications. J. Electromagn. Eng. Sci..

[2] Zhang F., Liu L., Zhang Y., Zhang F. (2024). Compact ultrathin wideband pattern-reconfigurable antenna with enhanced operating bandwidth. IEEE Antennas Wirel. Propag. Lett..

[3] Kahar M., Mandal M.K. (2021). A wideband tightly coupled slot antenna for 360° full azimuthal beam steering applications. IEEE Trans. Antennas Propag..

[4] Haydhah S., Ferrero F., Lizzi L., Sharawi M.S., Zerguine A. (2023). Multifunction pattern reconfigurable slot-antenna for 5G sub-6 GHz small-cell base-station applications. IEEE Access.

[5] Haydhah S.A., Ferrero F., Lizzi L., Sharawi M.S., Zerguine A. (2021). A multifunctional compact pattern reconfigurable antenna with four radiation patterns for sub-GHz IoT applications. IEEE Open J. Antennas Propag..

[6] Darvazehban A., Ahdi Rezaeieh S., Manoochehri O., Abbosh A.M. (2020). Two-dimensional pattern-reconfigurable cross-slot antenna with inductive reflector for electromagnetic torso imaging. IEEE Trans. Antennas Propag..

[7] Wang W., Liujia E., Zhang J., Mei Z. (2025). A four-beam switchable cascaded cavity antenna loaded with bifunctional slots. Int. J. Electron. Commun..

[8] Bhattacharjee A., Dwari S. (2021). A monopole antenna with reconfigurable circular polarization and pattern tilting ability in two switchable wide frequency bands. IEEE Antennas Wirel. Propag. Lett..

[9] Jin X., Liu S., Yang Y., Zhou Y. (2022). A frequency-reconfigurable planar slot antenna using S-PIN diode. IEEE Antennas Wirel. Propag. Lett..

[10] Zhou C., Wang B., Wong H. (2021). A compact dual-mode circularly polarized antenna with frequency reconfiguration. IEEE Antennas Wirel. Propag. Lett..

[11] Behdad N., Sarabandi K. (2006). A varactor-tuned dual-band slot antenna. IEEE Trans. Antennas Propag..

[12] Kim J.P., Park W.S. (1998). Network modeling of an inclined and off-center microstrip-fed slot antenna. IEEE Trans. Antennas Propag..

[13] Shahrbandian M., Aliakbarian H. (2021). Removing broadside null of symmetric long-slot antennas by using a coating layer. IET Microw. Antennas Propag..

[14] Li E.S., Cheng J.-C., Lee S.-C., Kuo Y.-H. (2018). Beam-switching antenna implemented by reconfigurable slots on cylindrical cavity. IET Microw. Antennas Propag..

[15] Behdad N., Sarabandi K. (2006). Dual-band reconfigurable antenna with a very wide tunability range. IEEE Trans. Antennas Propag..

[16] Pozar D.M. (2004). Microwave Engineering.

[17] Janaswamy R., Schaubert D.H. (1986). Characteristic impedance of a wide slotline on low-permittivity substrates. IEEE Trans. Microw. Theory Tech..

